# Pathway Tools Visualization of Organism-Scale Metabolic Networks

**DOI:** 10.3390/metabo11020064

**Published:** 2021-01-22

**Authors:** Suzanne Paley, Richard Billington, James Herson, Markus Krummenacker, Peter D. Karp

**Affiliations:** 1 Bioinformatics Research Group, SRI International, Menlo Park, CA 94025, USA; paley@ai.sri.com (S.P.); billingt@ai.sri.com (R.B.); kr@ai.sri.com (M.K.); 2 Robotics Group, SRI International, Menlo Park, CA 94025, USA; james.herson@sri.com

**Keywords:** metabolic charts, metabolic diagrams, metabolic network diagrams, biochemical pathways charts, metabolic maps, transcriptomics, metabolomics

## Abstract

Metabolomics, synthetic biology, and microbiome research demand information about organism-scale metabolic networks. The convergence of genome sequencing and computational inference of metabolic networks has enabled great progress toward satisfying that demand by generating metabolic reconstructions from the genomes of thousands of sequenced organisms. Visualization of whole metabolic networks is critical for aiding researchers in understanding, analyzing, and exploiting those reconstructions. We have developed bioinformatics software tools that automatically generate a full metabolic-network diagram for an organism, and that enable searching and analyses of the network. The software generates metabolic-network diagrams for unicellular organisms, for multi-cellular organisms, and for pan-genomes and organism communities. Search tools enable users to find genes, metabolites, enzymes, reactions, and pathways within a diagram. The diagrams are zoomable to enable researchers to study local neighborhoods in detail and to see the big picture. The diagrams also serve as tools for comparison of metabolic networks and for interpreting high-throughput datasets, including transcriptomics, metabolomics, and reaction fluxes computed by metabolic models. These data can be overlaid on the metabolic charts to produce animated zoomable displays of metabolic flux and metabolite abundance. The BioCyc.org website contains whole-network diagrams for more than 18,000 sequenced organisms. The ready availability of organism-specific metabolic network diagrams and associated tools for almost any sequenced organism are useful for researchers working to better understand the metabolism of their organism and to interpret high-throughput datasets in a metabolic context.

## 1. Introduction

Synthetic biologists, metabolomics researchers, and microbiome researchers increasingly demand information regarding organism-scale metabolic networks. Conversely, the convergence of genome sequencing with computational methods for metabolic network reconstruction have yielded organism-scale metabolic network information for thousands of sequenced organisms. These networks are large, complex, and highly interconnected, and are therefore challenging for scientists to comprehend. Visualizing metabolic networks accelerates scientific understanding and analysis, and enables scientists to perform numerous network-based analysis operations, such as comparing the networks of one or more organisms, and interpreting omics datasets.

We present a rich set of bioinformatics tools that are available within the Pathway Tools (PTools) software for generating and manipulating organism-scale metabolic network diagrams. The simplest versions of these metabolic charts depict the full metabolic network of a unicellular organism. The charts have also recently been extended to depict the different subnetworks that are active in the different cell types of multi-cellular organisms. These charts have also been extended to depict the pan metabolic network computed from a pan-genome and to illustrate the metabolic networks of an organism community. Pathway Tools’ metabolic charts are also used to visualize comparisons of genome-scale metabolic networks and serve as tools for interpreting high-throughput datasets, including transcriptomics data, metabolomics data, and metabolic fluxes computed by metabolic models. These tools have been evolving for several years and have recently been re-engineered for faster operation with higher quality graphics within modern web browsers.

The remainder of the Introduction summarizes our past work on PTools metabolic charts including their graphical organization and generation. That past work lays the foundation for the recent enhancements presented in the Results section. The new implementation is JavaScript-based for speed and improved graphics quality. It provides fast semantic zooming whose most detailed level identifies metabolites, genes, enzymes, and pathways. The new diagram gives animated views of omics data with a dynamically controlled color scale.

### 1.1. Pathway Tools Background

PTools [1,2] is an extensive bioinformatics software system whose capabilities include genome informatics, pathway informatics, omics data analysis, and metabolic modeling. Its pathway informatics capabilities include metabolic reconstruction, metabolic route search, and quantitative metabolic modeling. Pathway reconstruction infers the metabolic reactions and pathways of an organism from its annotated genome and produces as output a Pathway/Genome Database (PGDB) containing the genome, proteome, reactome, and metabolic pathways of the organism. PTools runs as a desktop application on Windows, MacOS, and Linux; the same binary executable also runs as a web server on MacOS and Linux. Most capabilities described here are available in both web and desktop modes; but some, such as the new JavaScript-based zooming capabilities, are present only on the web, while others, such as the new community overview capabilities, are currently present only in desktop mode. PTools powers 19 bioinformatics websites [3] including BioCyc.org [4].

### 1.2. Past Work on Pathway Tools Metabolic Network Diagrams

PTools metabolic charts have evolved through multiple generations of improvements [5,6,7,8,9,10,11,12]. Early versions were manually drawn, contained the metabolic network only, and were available through the desktop version of PTools only. Later versions were constructed by automated layout algorithms, contained additional cellular structures such as the inner and outer membrane and periplasm, and were available via both desktop PTools and its web server mode. Additional labels were added as the layouts improved. The initial web version of the diagrams had no zooming; a later version implemented zooming through multiple levels of tiled images, thus yielding several discrete zoom levels whose information content did change (semantic zooming), although downloading of the tiled images was fairly slow. Omics data painting and omics pop-ups were not present in the early versions, and were added in later versions.

### 1.3. Organization of Pathway Tools Metabolic Charts

**Figure 1:**
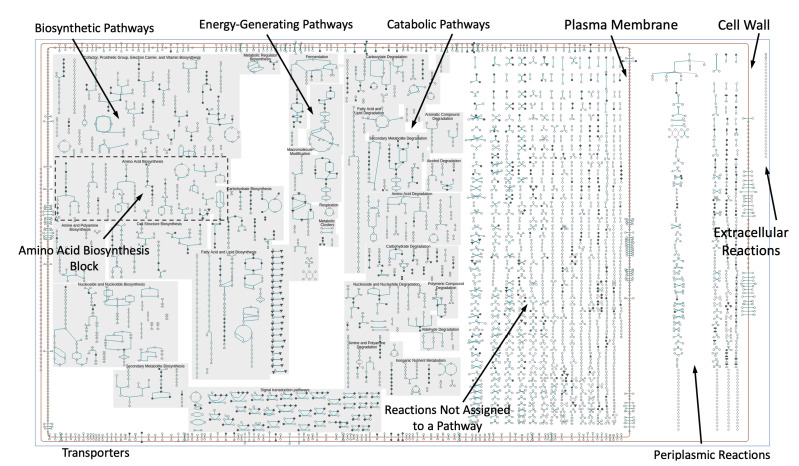
Components of the metabolic chart for *E. coli* K–12 (online diagram: [13]).

Each PTools metabolic chart, also known as a cellular overview diagram, is organized according to the cellular architecture of the organism (that architecture is described within the PGDB), depicting the cytosol, extracellular space, and plasma membrane. For Gram-negative bacteria, an additional outer membrane and periplasm are included (see Figure 1, online diagram for *Escherichia coli* is at Reference [13]). Within the cytoplasm, metabolic pathways are shown to the left side, and reactions not assigned to any pathways are shown to the right. Within the pathway area, biosynthetic pathways are drawn to the left, energy metabolism pathways in the middle, and degradation pathways on the right. Within these areas are blocks of pathways organized according to the MetaCyc pathway ontology (e.g., the biosynthesis area contains blocks called Carbohydrate Biosynthesis and Fatty Acid and Lipid Biosynthesis). Pathways flow downward within the charts.

Transporters and other membrane proteins are drawn on the appropriate membrane, and reactions or proteins located in the periplasm or extracellular space are drawn between or outside the membranes. Lines in the chart depict biochemical reactions, and nodes depict metabolites. Nodes are shape coded, for example, to distinguish amino acids from carbohydrates from tRNAs.

Connections between pathways are not shown to keep the diagram from becoming unduly tangled, except insofar as pathways are combined into superpathways that are defined in the PGDB. Using the desktop PTools, the user can interactively add selected connections between metabolites to explore the connectivity of the network. A PDF version of the chart for any organism, which can be printed as a poster, is available at BioCyc.org via the command Metabolism→Generate Metabolic Map Poster.

### 1.4. Creation of Pathway Tools Metabolic Charts

The input required to construct a PTools metabolic chart is the metabolic network of one or more organisms described as one or more PGDBs. Each PGDB can capture the genome, proteome, metabolome, reactome, and metabolic pathways of an organism. PGDBs in turn are created by the PathoLogic module of PTools, which computes a metabolic reconstruction from a user-supplied annotated genome. All metabolic charts in this article were generated from PGDBs obtained from BioCyc. BioCyc PGDBs were generated from GenBank annotations with varying degrees of subsequent manual curation.

Most BioCyc metabolic charts are organism-specific in the sense that they include only the pathways, reactions, enzymes, genes, and metabolites present in a single organism’s PGDB. When the chart is regenerated, it automatically includes the latest information from the organism’s PGDB, such as reactions or pathways that curators have deleted or added using the PTools editors [2].

PTools creation of a new diagram follows the following procedure.

- Query the PGDB to determine the cellular architecture of the organism, meaning what membranes and cellular spaces are present
- Query the PGDB to determine the sets of reactions, proteins, and pathways present in different regions of the diagram, such as the sets of biosynthetic, catabolic, and energy-related pathways
- Lay out each individual pathway diagram using PTools pathway layout algorithms [14]
- Run a layout algorithm that arranges the pathways within each pathway block (such as Amino Acid Biosynthesis)
- Run a layout algorithm that arranges the pathway blocks relative to one another within the biosynthesis, catabolism, and energy-metabolism regions
- Lay out the grids of individual reactions in the cytosol, periplasm (if applicable), and extracellular space
- Arrange the transporters in the cellular membrane(s)

## 2. Results

### 2.1. New JavaScript Implementation of Metabolic Charts

We recently re-engineered the web version of PTools charts to overcome several limitations of the previous implementation. The previous implementation used sets of tiled images at different zoom levels that were generated on the server side. The diagram was slow to zoom (zooming did not feel real time) because the tiled images were slow for the web browser to download from the server. Also problematic was the existence of discrete zoom levels for the diagram—the user could not obtain a zoom level in between those pre-specified levels.

**Figure 2:**
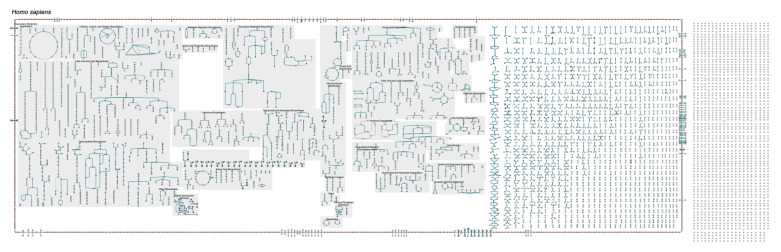
The complete human metabolic chart, at its lowest zoom level (online diagram: [15]). HumanCyc version 24.0 contains 347 metabolic pathways.

In the new implementation (see Figure 2) the graphics behind each chart are still drawn using Common Lisp graphics operations in the PTools web server so that we can utilize the existing layout algorithms. However, those graphics are translated to a JSON representation of the graphics operations and then downloaded to the user’s web browser. Once the JSON representation of the graphics have been downloaded from the server, they are rendered to a canvas within the web browser by a JavaScript package we developed called WG. The rendering and zooming occur within the user’s browser and are quite fast—zooming of the diagram occurs in real time. The JSON representation is generated once for each organism and is saved as a file that is cached on the server side. Most of our figure legends include URLs that will re-create live versions of the charts shown in each figure. Firefox and Chrome are recommended web browsers for BioCyc.org.

**Figure 3:**
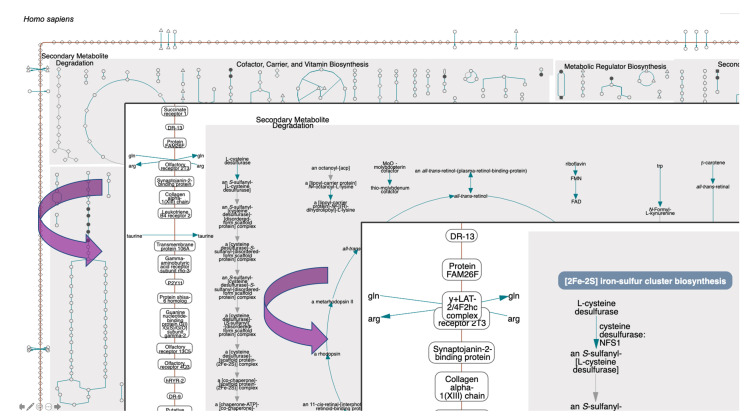
The upper left corner of the human metabolic chart (online diagram: [15]) is shown at three semantic zooming levels. Level 1: Pathway block labels only. Level 2: Addition of metabolite labels and labels for transporters and membrane proteins. Level 3: Addition of gene and enzyme labels.

To achieve smooth semantic zooming (see Figure 3), four discrete versions of the diagram are generated on the server side, at four different sizes. Each level contains different information content, for example, the highest detail level includes metabolite, gene, and enzyme labels, but the lowest detail level contains none of those labels. However, to the user discrete levels are not apparent; instead, as the user rotates the mouse wheel to enlarge the diagram, the diagram for the current level smoothly increases in size. Once its size matches the next semantic zoom level, that next level is drawn in its entirety, with the additional detail visible. At the lowest zoom level, metabolites are shown as icons whose shape corresponds to their chemical class (e.g., triangles for amino acids, squares for carbohydrates), and the only visible labels are the pathway class labels. At higher magnification levels, the metabolite icons are replaced with labels, then individual pathway labels appear, and finally the enzyme and gene names become visible.

**Figure 4:**
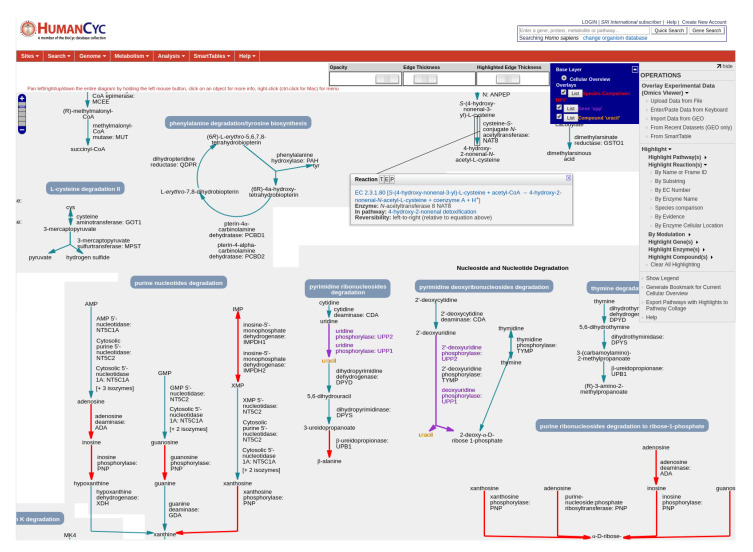
A portion of the human metabolic chart (online diagram: [15]), shown at its highest zoom level with all labels drawn, and displaying a reaction tooltip. Three highlighting operations have been performed. (1) A species-comparison operation highlights all reactions shared with *Helicobacter pylori* in red. (2) All genes whose names include the substring UPP are colored purple. (3) The compound uracil has been highlighted in orange.

Tooltips provide further details when the user mouses over a metabolite or a reaction, such as the reaction equation, EC number, enzyme name(s), and containing pathway(s). Figure 4 shows a portion of the human metabolic chart at the highest magnification level.

### 2.2. Search and Highlight Operations

**Figure 5:**
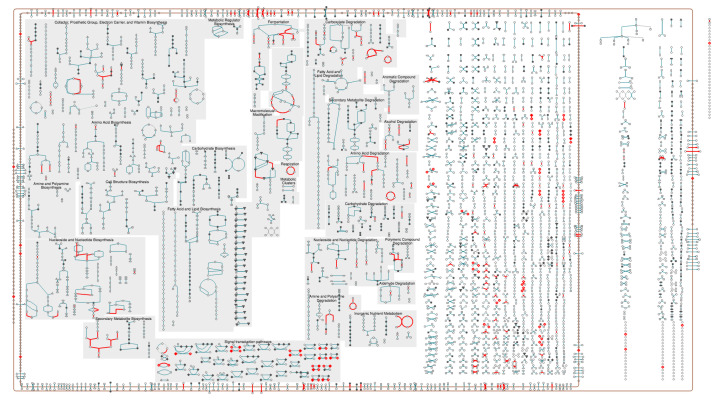
The metabolic chart for *Escherichia coli* K–12 (generated from BioCyc.org); highlighted in red are all reactions catalyzed by genes in the regulon of FNR, a transcriptional regulator that mediates the transition from aerobic to anaerobic growth.

Search operations are available in the right-sidebar menu to locate genes, metabolites, reactions and pathways of interest within a chart. For example, the user can search for reactions by name, identifier, substring, EC number, enzyme name, evidence code, and by cellular location of the enzyme. In addition, users can search by regulation (e.g., find all enzymes with a particular activator, inhibitor, or cofactor; or all genes in the regulon of a particular transcription factor), or by curation level (e.g., all pathways or enzymes with citations, comments, and/or experimental evidence). The Species Comparison operation highlights all reactions shared or not shared with one or more other selected organisms. Figure 4 shows the results of three example highlighting operations on a portion of the human metabolic chart. Figure 5 shows the *E. coli* metabolic chart highlighting all genes directly regulated by FNR, a transcriptional regulator that mediates the transition from aerobic to anaerobic growth.

Because BioCyc charts are so information-dense, visually picking out all highlighted entities can be difficult. In the web implementation, we offer two solutions. Sliders at the top of the page enable the user to decrease the opacity of non-highlighted portions of the diagram and/or increase the width of highlighted reaction edges relative to non-highlighted edges. Either individually or in combination, these simple adjustments make the highlighted entities stand out visually. Alternatively, the user can request a list of all entities highlighted in a particular operation and then select one to identify with a distinct marker icon.

A particular metabolite may appear in multiple different reactions, and a particular reaction may appear in multiple pathways within a chart. In the desktop implementation, right-clicking on a compound node or reaction edge brings up a menu of operations, including navigating to the detail page for that compound, reaction or pathway either in the main display or in a pop-up window, and highlighting the compound, reaction, pathway, or all reactions of the compound everywhere they appear within the diagram. One option in the compound menu is to draw connections from that compound node to other instances of that compound elsewhere in the chart. A control panel enables the user to selectively hide or show subsets of possible connections.

### 2.3. Cell-Type-Specific Metabolic Charts

**Figure 6:**
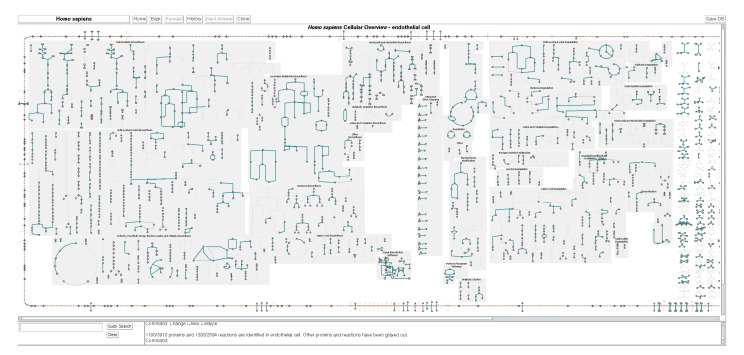
The metabolic chart for a human endothelial cell (generated by desktop PTools). Reactions and proteins for which there is no evidence in this cell type are grayed out but remain faintly visible. The pane at the bottom lists the number of metabolic and transport proteins and reactions that are present in this cell type as a fraction of the total metabolic network.

A new visualization, currently available only in the downloadable PTools software running in desktop mode, depicts the metabolic chart customized for the metabolic reactions and pathways active in specific cell types. When a PGDB captures a representation of multiple different cell types, each protein can be annotated with the set of cell types in which it has been observed. We have defined six cell types in the HumanCyc database: endothelial cell, hepatocyte, leukocyte, oocyte, sperm, and type B pancreatic cell. These cell types were selected because protein data for them is available and could be imported from the Bgee database of gene expression patterns [16]. When visualizing a given cell type, the chart queries the set of proteins active in that cell type based on the Bgee data, and then computes the active-reaction complement of that cell type as the set of reactions catalyzed by the active proteins. The chart grays the reactions of the complete metabolic network that are not active in that cell type. An example metabolic chart for a human endothelial cell is shown in Figure 6.

### 2.4. Metabolic Charts for Pan-Genomes

Although most metabolic charts include pathways, reactions and enzymes for a single organism, BioCyc also includes several pan-genome PGDBs. These databases contain the union of all genes, enzymes, reactions, and pathways for all strains of a particular species for which annotated genomes are present in BioCyc. Thus, the metabolic chart for a pan-genome PGDB illustrates the full range of metabolic and transport functionality across members of the species. Pan-genome databases are currently available in BioCyc for nine medically important and well-studied species: *Clostridioides difficile*, *Escherichia coli*, *Helicobacter pylori*, *Listeria monocytogenes*, *Mycobacterium tuberculosis*, *Pseudomonas putida*, *Salmonella enterica*, *Shigella flexneri*, and *Vibrio cholerae*.

A pan-genome PGDB is constructed as follows. A new, empty PGDB for a species is created. We select a so-called “lead PGDB” from the set of available strain-specific PGDBs. It is usually the earliest sequenced, most experimentally characterised, and best annotated strain. All of the lead organism’s replicons, genes, proteins, reactions, and pathways are imported into new the pan-genome PGDB. Thereafter, all remaining strain PGDBs from the chosen set are visited, and each protein-coding gene in that PGDB is checked for orthology with the genes in the pan-genome PGDB. If an ortholog is found, the software records the existence of the ortholog in the gene of the pan-genome PGDB. If no ortholog is found, then the gene is imported from the strain PGDB, along with its proteins and any reactions and pathways associated with that gene that are not yet in the pan-genome PGDB.

**Figure 7:**
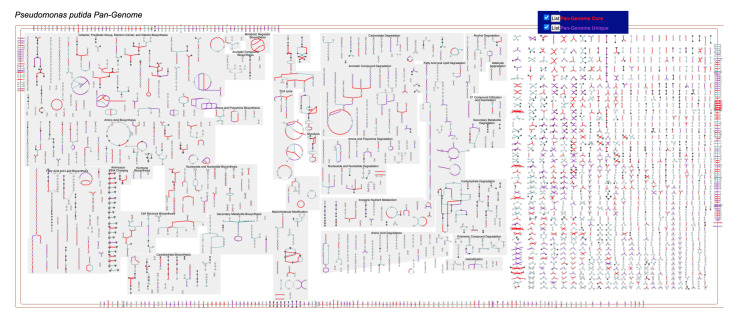
The complete metabolic chart for the *Pseudomonas putida* pan-genome (online diagram: [17]). Reactions catalyzed by genes in the core genome are highlighted in red. Reactions catalyzed by genes that are unique to a single species are highlighted in purple.

Two additional search options are available for pan-genome metabolic charts. Under the web Highlight Gene(s) menu are commands to highlight either pan-genome core genes or pan-genome unique genes. Core genes are those for which orthologs exist in all strains of the species. Thus, this highlight command illustrates the metabolic and transport functionality that is a fundamental characteristic of the species. Unique genes are those that only exist in a single strain. Note that some reactions will be highlighted both as core and unique if they have multiple isozymes, one of which is part of the core genome and another of which is unique to one strain. Figure 7 shows the metabolic chart for the *Pseudomonas putida* pan-genome, with both core and unique genes highlighted.

### 2.5. Metabolic Charts for Organism Communities

**Figure 8:**
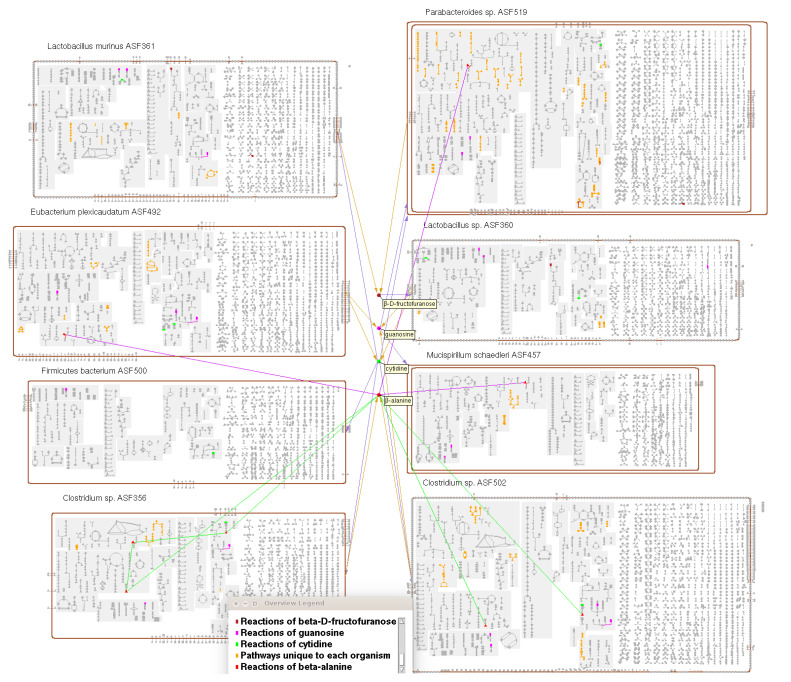
A community overview diagram for the eight bacterial species that make up the altered Schaedler flora model gut microbiome [18] (generated by desktop PTools). Four potential exchange metabolites have been included, and the reactions of each of these metabolites have been highlighted in a different color, as shown in the legend. For one of these metabolites, beta-alanine, connections have been drawn to all instances of this metabolite in each organism. Pathways that are unique to each organism have been highlighted in orange.

A new visualization, currently only available in the downloadable PTools software running in desktop mode, is the community overview diagram, which combines the metabolic charts for multiple selected organisms, along with optionally one or more exchanged metabolites, into a single display (see Figure 8). The metabolic diagram for each organism is scaled down, and all of the diagrams are displayed together in two columns, with exchanged metabolites in a middle column, with connections drawn to the producing and consuming organism(s) for each metabolite. We envision two possible use cases for the diagram. It can be used to visually explore metabolic interactions among a community of interacting or coexisting organisms, such as might be found in a metagenomic sample, including by overlaying omics data. Alternatively, it can be a tool for comparing the metabolic and transport complement of multiple related (but not necessarily interacting) organisms.

The user must specify the set of exchange metabolites to be included, if any. If particular metabolites are known to be exchanged, they can be specified explicitly, along with which organisms in the community import and export those metabolites. Alternatively, the user can request that the software suggests possible exchange metabolites. Suggestions are based on the presence of influx or efflux transporters for a metabolite, and/or the identification of metabolites that are consumed but not produced in a dead-end metabolite analysis.

Most of the same search and highlight operations that are available for BioCyc single-organism metabolic charts have been extended to also support searching and highlighting across all the organisms that make up a community overview. Many of these highlight operations give the user the choice of applying them to all of the organisms in the community or to just one or a subset of them.

Combining metabolic charts for multiple organisms into a single diagram facilitates new species-comparison operations. We have added four new highlighting operations: reactions present in all organisms, pathways present in all organisms, reactions unique to a single organism, and pathways unique to a single organism. A pathway is considered present in an organism so long as some variant of it is present. For example, one organism might have a different variant of an arginine degradation pathway than some other organism—for the purposes of this comparison, these are not considered unique pathways.

### 2.6. Omics Data Analysis with Metabolic Charts

**Figure 9:**
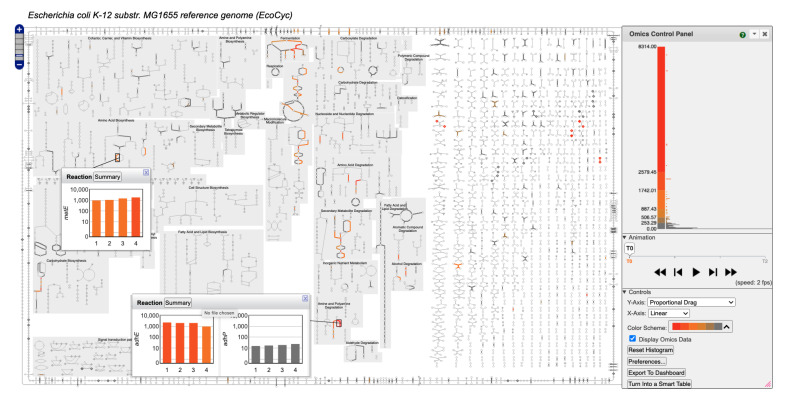
The *Escherichia coli* metabolic chart overlaid with a gene-expression time series dataset [20] showing growth during a transition from anaerobic to aerobic growth (online diagram: [21]). The control panel on the right enables the user to either step through the individual time points or play them as an animation. It also enables the user to choose from multiple color scheme options and to manually adjust the value ranges for each color. The histogram to the right of the color scale shows the distribution of data values for the current timepoint. Popup bar graphs are displayed for two reactions, showing how their gene expression changes over the course of the experiment.

A PTools metabolic chart can be used as a vehicle for metabolism-based interpretation of omics data, such as gene expression or metabolomics data. In this mode of use the chart color codes reactions and metabolites according to omics data values provided in an input file, and enables the user to see which pathways and reactions are more or less active under some set of experimental conditions. The tool automatically maps the omics data range to a user-selected color scale. A data series that consists of multiple time points or conditions can be viewed as an animation. In the web implementation, the control panel to the right of Figure 9 enables selection among several color scales, and alteration of the mapping from omics values to that color scale. For example, the dataset shown in Figure 9 contains a few very large data values that by default map to red at the top of the scale; virtually all the other genes are colored gray at the bottom of the scale. But by clicking and dragging the y-axis labels in the color scale the user can adjust the size of each color bucket to enable coloring of a wider range of data values. The user can also change the width of colored edges and the opacity of uncolored portions of the diagram to visually emphasize the colored edges. A short video describing chart-based omics analysis is available at Reference [19].

Users can input omics data to this tool in several ways. Data can be uploaded from a tab-delimited file, from a SmartTable, or imported directly from GEO [22] curated datasets. Using the desktop PTools, metabolomics data can also be imported from Metabolomics Workbench [23]. During data import our software attempts to identify every input metabolite using metabolite names and identifiers present in BioCyc PGDBs, including identifiers from BioCyc, KEGG, PubChem, MetaNetX, ChEBI, SEED, ChemSpider, HMDB, MetaboLights, and others.

When transcriptomics or proteomics data are displayed, mousing over a reaction (or mousing over a metabolite when metabolomics data are shown) in a chart and clicking the Omics button in the resulting tooltip window generates an “omics-popup” window that graphs the data for the relevant objects over the entire data series. The user can choose whether this data is shown as a bar graph, an X-Y plot, or a heatmap. Omics data can be overlaid onto a community overview diagram, just as for a single-organism diagram. Users specify which of the organisms in the community have available omics datasets and supply the data for each organism in a separate file.

This tool is part of a full suite of omics analysis and visualization tools available in PTools. In addition to displaying omics data on metabolic charts, data can be viewed (1) on individual pathway diagrams; (2) on a table of the *N* most highly perturbed pathways based on a pathway-perturbation score calculated from the omics data; (3) on a user-specified set of pathways (a Pathway Collage); (4) on a genome-scale regulatory network diagram called the regulatory overview [24]; and (5) on the Omics Dashboard. These tools complement one another, facilitating exchange of data between them, and providing multiple entrees into the data, both at the highest levels of organism function, and focusing in on more specific areas of interest.

### 2.7. Integration with Other Modules of Pathway Tools

PTools supports a variety of other analysis tools, many of which are tightly integrated with metabolic charts, making visualizing analysis results from multiple biological perspectives easy. Tools integrated with metabolic charts include the following:

- SmartTables [25] enable users to generate or upload data such as a list of genes or metabolites to a “knowledge spreadsheet” and then manipulate it in various ways. Entities from one or more SmartTable columns can be painted as sets of highlights onto the metabolic chart for the appropriate organism. SmartTables containing omics data can be painted onto a metabolic chart. Alternatively, a set of highlighted objects in the metabolic chart can be exported to a new SmartTable.
- MetaFlux [26] enables construction of metabolic models using flux-balance analysis. Users can either create their own metabolic models using the desktop PTools or can run existing models within the BioCyc website (e.g., for *E. coli*). The steady state reaction fluxes computed by a model can be painted onto the metabolic chart for that organism, to visualize the activity levels of different portions of the metabolic network under the modeled conditions.
- A dead-end metabolite analysis tool generates lists of metabolites that are either consumed but not produced, or produced but not consumed by any reaction in an organism’s metabolic network. These metabolites represent either true cellular inputs and outputs, or gaps in the metabolic network corresponding to missing information. The metabolite lists generated by such an analysis can be highlighted on the metabolic chart.
- A chokepoint analysis tool identifies reactions that are potential drug targets because they represent chokepoints in the metabolic network, such that eliminating them would eliminate the only path to or from a particular metabolite. These reactions can be highlighted on a metabolic chart.
- The Pathway Collage Viewer [27] enables generation and editing of personalized multi-pathway diagrams called Pathway Collages. Pathway Collages bridge the gap between the low-level view of a single pathway and the high-level global view of the entire metabolic network by enabling users to select specific pathways of interest to combine into an intermediate-level diagram. Pathway Collages can be customized in various ways, and optionally overlaid with omics data. Users who have used the full metabolic chart to highlight one or more objects of interest can invoke the Pathway Collage Viewer to generate a collage of all pathways that either have themselves been highlighted or for which some component gene, reaction, or metabolite has been highlighted. In addition, when uploading omics data to a metabolic chart, users can choose between displaying data on the full metabolic chart or on a newly generated Pathway Collage consisting of only the N most perturbed pathways.
- The Omics Dashboard [28] is a novel tool for interactively exploring omics data through a hierarchical set of graphs organized around cellular systems. At its highest level, the Omics Dashboard contains panels for high-level systems such as Biosynthesis and Regulation, with plots for each of their subsystems, such as Amino Acid Biosynthesis and Cofactor Biosynthesis, that amalgamate the omics data values for all genes or metabolites in that subsystem. Drilling down on an individual subsystem such as Amino Acid Biosynthesis shows plots for the biosynthesis pathways of each individual amino acid. Users can continue to drill down on subsystems of interest until they reach the level of individual genes or metabolites. The Omics Dashboard offers a different, complementary view of omics data than do metabolic charts. From a metabolic chart painted with omics data, users can click a button to open a new tab with the data displayed on the Omics Dashboard and thus have access to both views simultaneously.

## 3. Discussion

In this section we compare PTools metabolic charts with those from several other tools: KEGG Mapper [29], KEGG Atlas [30] (website defunct), iPath 2.0 [31], and Escher [32]. All of the four tools use manually drawn diagrams that therefore cannot be updated automatically when the underlying metabolic database changes. None of the other tools provides semantic zooming, which is quite problematic for KEGG Mapper, KEGG Atlas, and iPath, as their diagrams lack labels for metabolites and enzymes, and thus identifying diagram elements requires multiple mouse clicks per metabolite or enzyme. KEGG Mapper, KEGG Atlas, and iPath all use static diagrams that combine pathways from multiple organisms rather than organism-specific diagrams; thus, a large portion of a given chart contains reactions and metabolites not found in the current organism. The other tools do not include transporters, or distinguish the periplasm or extracellular space; they do not depict the pathways found in different cell types nor depict charts for organism communities. All of the tools produce metabolic-network diagrams that can be painted with omics data; however, none enables dynamic user control over the color scale. None of the other tools provides omics pop-ups. Only iPath can produce animated visualizations of omics data.

## 4. Conclusions

PTools metabolic charts have been developed over many years and have grown from a manually drawn diagram for a single organism to automatically formatted diagrams for more than 18,000 organisms. The charts depict which pathways are active in different cell types and can be used to analyze pan-genomes and organism communities. PTools charts are viewable online and printable as posters. Their myriad search operations enable users to rapidly locate enzymes, pathways, and metabolites of interest, and to compare the metabolic networks of different organisms. PTools metabolic charts are tightly integrated with other modules of PTools including quantitative metabolic models, SmartTables, computation of dead-end metabolites and metabolic choke points, and with Pathway Collages and the Omics Dashboard. A new JavaScript-based implementation of BioCyc charts provides higher-quality graphics and enables real-time semantic zooming of the diagrams and animated overlay of omics data with dynamic control of the color scale.

## 5. Availability and Requirements

Project name: Pathway Tools.

Project home page: https://brg.ai.sri.com/ptools/.

Operating systems: Linux, MacOS and Windows.

Programming languages: Common Lisp and JavaScript.

License: Pathway Tools is free to academic users, and is available for a fee to commercial users. To obtain the software, see https://BioCyc.org/download.shtml.

## Data Availability

The BioCyc data used to generate BioCyc metabolic charts are freely and openly available via https://BioCyc.org/download.shtml.
